# Between support and uncertainty: employers’ perspectives on workplace inclusion of people with mental health problems

**DOI:** 10.1186/s12913-026-15665-x

**Published:** 2026-09-28

**Authors:** Silvia Krumm, Lea Mayer, Sarah Feige, Nicole Stasch, Lena Renz, Christiane Braun-Volkert, Reinhold Kilian, Edith Almer

**Affiliations:** 1 Department for Psychiatry and Psychotherapy II, Ulm University, Ulm, Germany; 2 Clinic and Policlinic for Psychiatry and Psychotherapy, Leipzig University, Leipzig, Germany; 3 MutMacherMenschen gemeinnützige eG, Augsburg, Germany; 4 MutMacherMenschen e.V., Augsburg, Germany

**Keywords:** Mental disorders, Labour market participation, Employers, Workplace, Stigma, Disclosure, Qualitative research

## Abstract

**Background:**

Despite increasing awareness of mental health in the workplace, people with mental health problems continue to face substantial barriers to labour market participation. While stigma-related explanations have been widely addressed, less is known about how employers respond to mental health problems within the constraints of everyday organisational practice. This study explores employers’ perspectives on workplace inclusion, with a particular focus on ambivalences, facilitating orientations, and expectations towards employees with mental health problems.

**Methods:**

This study reports findings from the first, qualitative phase of a participatory mixed-methods research project conducted in Southern Germany. Between November 2022 and August 2023, we conducted 29 semi-structured interviews with employers from companies across different sectors. Data were analysed using structured qualitative content analysis.

**Results:**

Employers described ambivalent orientations towards the inclusion of people with mental health problems. While mental health issues were seen as increasingly normalised and framed within broader societal developments, employers reported uncertainties regarding work organisation, team functioning, and predictability. Facilitating orientations included social responsibility, recognition of the health-promoting role of employment, pragmatic responses to labour shortages, and positive prior experiences. At the same time, workplace participation was often described as depending on employees’ transparency regarding work-related limitations, individual responsibility for managing mental health, and disclosure as a prerequisite for employers’ support and mutual trust.

**Discussion:**

Workplace inclusion of people with mental health problems appears to depend not only on employers’ attitudes but also on practical considerations related to work organization and team functioning. Employers’ perspectives reflected ambivalence, balancing commitments to participation with concerns about predictability, fairness, and collective functioning. Expectations regarding disclosure, responsibility, and transparency were central to perceived feasibility of support.

**Conclusions:**

Addressing these organisational dynamics may help inform more context-sensitive strategies to promote sustainable labour market participation.

**Supplementary Information:**

The online version contains supplementary material available at https://doi.org/10.1186/s12913-026-15665-x.

## Background

Competitive employment is a central dimension of social participation in contemporary societies. Beyond providing financial resources, it fulfils important latent functions, including structuring everyday life, enablinonstituteg social integration, and contributing to identity and purpose [1]. Employment is associated with improved well-being and mental health, greater autonomy, enhanced social status, and opportunities for personal development [2]. Research indicates a reciprocal relationship between unemployment and mental health, as job loss contributes to psychological distress while pre-existing mental health problems simultaneously increase the risk of job loss and prolonged unemployment [3]. This interplay places people with mental health problems at particular risk of cumulative disadvantage, as exclusion from employment reinforces economic hardship, social isolation, and poorer health outcomes [4]. Despite growing policy attention and an expanding evidence base emphasising the importance of labour market participation, people with mental health problems continue to experience substantial barriers to employment [5]. In Germany, for example, a recent multicentre study found that only around one-third (34%) of individuals with severe mental illness (SMI) are engaged in competitive employment [6].

Barriers to labour market participation are not only related to individual impairments that may temporarily limit work capacity, but are also shaped by broader social and organisational processes, including stigma and discriminatory practices [7]. A systematic review has demonstrated that stigma contributes to reduced hiring opportunities, lower income, and discrimination following disclosure of mental health problems [8]. More recent meta-analytic findings indicate persistent discrimination, particularly towards people living with psychosis, often linked to concerns about unpredictability, productivity, and workplace safety [9]. Experimental and survey-based studies further suggest that disclosure of a mental illness may lead to more cautious employer evaluations and reduced hiring prospects despite comparable qualifications [10–13]. In addition, employers’ uncertainties regarding legal frameworks and limited organisational preparedness have been identified as potential barriers to inclusive employment practices [14]. Field experimental research has also shown significantly lower interview invitation rates for applicants disclosing a history of mental health problems [15]. Qualitative studies point to the perception that demanding work environments may be incompatible with mental health problems, while prior negative experiences can reinforce stereotypical assumptions [16].

While stigma-related barriers have received considerable attention, less is known about how employers interpret, negotiate, and operationalise workplace inclusion within the constraints of everyday organisational practice. Evidence suggests gradual improvements in organisational awareness of mental health issues and a growing recognition of the value of employment for recovery and social participation [14]. Recent research has also addressed the measurement of workplace integration of people with disabilities [17]. At the same time, qualitative research indicates that employers’ perspectives are often characterised by ambivalence, reflecting the need to balance normative commitments to inclusion with practical concerns about reliability, performance, and organisational feasibility [16, 18]. This tension suggests that workplace inclusion may be shaped not only by individual attitudes but also by practical and organisational considerations.

As organisational decision-makers, employers strongly influence how workplace inclusion is negotiated and implemented in everyday practice. However, little is known about employers’ expectations towards employees with mental health problems, how these expectations relate to organisational constraints, and how they are translated into workplace practices. Understanding these processes may help to explain how organisational conditions shape workplace inclusion.

Against this background, the present qualitative study examines employers’ perspectives by addressing the following research questions:

1. How do employers describe their perspectives on employees with mental health problems including both supportive and challenging aspects?
2. How do employers describe expectations and conditions for workplace support in organisational practice?

## Methods

The reporting of the study was guided by the Consolidated Criteria for Reporting Qualitative Research (COREQ).

### Study design

This study was conducted as part of the participatory mixed-methods study “TAPE – Promoting the participation of people with severe mental illness in the workplace. Attitudes, barriers, and facilitating factors” (2022–2025). The aim of TAPE was to identify the perspectives of employers and people with experiences of mental illness on facilitators and barriers to labour market participation in order to develop recommendations for employers. In the first step, qualitative interviews were conducted with employers and people with experience of mental illness about their labourmarket experiences, attitudes, and needs. The qualitative design was chosen to explore employers’ perspectives and the organisational contexts shaping decisions related to workplace participation. The qualitative findings served as the basis for the development of standardised questionnaires conducted in the second step, which were used in both study groups to examine the findings on a larger sample. The present study focuses on the results of the qualitative interviews with employers.

### Research team

In line with the participatory design, the research team was composed of people with and without experiences of mental illness. People with experience of mental illness were involved in the entire research process as co-researchers. Participatory research workshops were regularly held to integrate experiential and professional perspectives into the research process. Participants received training in research principles, including both quantitative and qualitative methods, interview techniques, and basic approaches to qualitative data analysis. Within the weekly held workshops, we developed interview guides, reflected preliminary findings, discussed interpretations, and iteratively refined the analytical categories.

### Study setting and recruitment

The study was conducted in Southern Germany and focused on small and medium-sized enterprises across different sectors. 29 employers and organisational decision-makers were recruited through regional networks, professional contacts, and collaboration with partner organisations involved in the broader research project. Purposive sampling was used to achieve variation in organisational roles, company size, sector, and prior experience with employees with mental health problems. Recruitment took place through various forms via existing networks and cooperation partners of the project (e.g., mailing lists, events), calls for study participation in regional and employer-related media, and direct written, telephone, or personal contact. Recruitment was challenging, likely due to the sensitivity of the topic, time constraints, and competing organisational priorities. Recruitment and analysis proceeded in parallel with some temporal overlap. Emerging codes and categories were regularly discussed within the research team throughout the analysis. Recruitment continued until subsequent interviews yielded repetitive findings and no new substantive codes or conceptual categories emerged. Based on this iterative process, we concluded that data saturation had been achieved.

### Participants

Participants were eligible if they held organisational decision-making responsibilities related to recruitment, supervision, or human resources. This included managing directors, department heads, and human resources professionals. Individuals without organisational decision-making responsibilities regarding recruitment, supervision, or personnel management were excluded. Purposive sampling was used to ensure diversity with regard to organisational roles, company characteristics, and prior experience with the workplace inclusion of people with mental health problems. Participant characteristics are presented in Table 1.

**Table 1:** Characteristics of participants (*n* = 29)

Position	
Owner / Management	12
Human Resources (various specialisations)	12
Occupational Physician	2
Management Position with Personnel Responsibility	2
**Industry**	
Manufacturing Industry, Mining, other Industries	7
Freelancers, scientific and technical	4
Public sector	3
Health and social services	2
Agriculture, forestry and fishing	3
Information and communication	2
Financial and insurance services	2
Social sector and health care	2
Other services	2
Trade, Transportation and Warehousing	1
**Gender**	
Female	18
Male	10
Not reported	1
**Company Size**	
Small	6
Medium	10
Large	12
**Experience**	
None	6
New hire + continued employment	10
Continued employment	12
New hire	12

### Data collection

Between November 2022 and August 2023, we conducted 29 semi-structured online interviews with employers, allowing for comparability across interviews while remaining open to new aspects. The interviews were conducted by two female researchers (SF and NS), who were experts by profession (psychology) and by experience. The interview guide was developed within the participatory research workshops, was informed by both the existing literature and experiential knowledge and covered topics including employers‘ experiences with employees with mental health problems, attitudes towards labour market participation, perceived facilitating and hindering factors, and expectations towards employees with mental health problems (See additional file 1). All interviews were audio-recorded, transcribed verbatim, and pseudonymised. All participants gave their informed consent. The interviews lasted between 21 and 59 min. Field notes were made after each interview to document contextual information and initial analytical reflections.

### Research team and reflexivity

The interviews were conducted by experts by profession and by experience. Interviewers were experienced in conducting semi-structured interviews or received training in qualitative interviewing techniques. No relationship was established between the interviewers and participants prior to the study. The participatory approach supported reflexivity by bringing different perspectives into discussions of potential assumptions and biases related to the study topic. Researchers with lived experience contributed to the development of interview guides, the discussion of emerging categories, and the interpretation of findings. Their perspectives informed reflexive discussions within the research team and helped to contextualise employers’ accounts from different experiential viewpoints.

### Data analysis

The qualitative data were analysed using structuring qualitative content analysis [19]. In a first step, all interviews were read in full to gain an overall understanding of the material. Based on the interview guide, initial main categories were developed deductively. The initial coding framework was iteratively refined during the coding process. Emerging categories and coding decisions were discussed regularly within the participatory research workshops until consensus on the category system was reached. Subsequently, relevant text segments were coded and additional subcategories were developed inductively from the data. New categories were continuously compared with previously coded material. The category system was iteratively refined through discussions within the participatory research workshops. Throughout the analysis, coded passages were repeatedly revisited to ensure consistency of category application across interviews. In a final step, coded segments were summarised and compared across cases to identify central categories. Data management and analysis were supported by MAXQDA 2024 software (VERBI, Berlin, Germany).

All interviews were conducted and analysed in German. For publication purposes, selected quotations were translated into English using a combination of researcher-based translation and AI-based language editing tools (deepL). Translations were reviewed within the research team for accuracy and consistency.

### Rigour

Coding took place within regular participatory research workshops and followed a consensual approach involving multiple researchers, with coding decisions discussed until consensus was reached. The development of the category system was systematically documented, and categories were iteratively condensed into higher-order categories. Consistent with the participatory research approach, preliminary findings were reflected upon within the project team and additionally presented in an overarching research workshop including researchers with qualitative methodological expertise and broad experiences in social psychiatric research.

### Ethics

In 2022, the study was approved by the Ethics Committee of the University of Ulm (180/22). All participants received written information about the study, including the interviewers’ background and role within the research project, and provided informed consent prior to participation. Data were anonymised during transcription and analysis to ensure confidentiality.

## Results

Three main categories were derived from the transcripts describing employers’ attitudes to employees with mental health problems: (1) ambivalences and uncertainties; (2) facilitating orientations and resource-focused perspectives, and (3) employers’ expectations towards employees with mental health problems.

### Between normalisation and uncertainty: ambivalent attitudes toward mental illness among employers

Employers express supportive attitudes and resource-oriented reasoning regarding the employment of employees with mental health problems. These relate in particular to 1. the normalisation of mental health problems, 2. uncertainties in dealing with mental health problems, 3. nuanced perceptions of diagnoses, and 4. specific challenges in the day-to-day work environment.

#### Normalisation of mental health problems

Employers frequently express supportive attitudes and use resource-oriented reasoning. Their statements reflect views that point to a growing normalisation of mental illness in both the workplace and society at large. Accordingly, mental illness can “affect anyone” and is increasingly viewed as a “normal” phenomenon in the workplace as well. It is “no longer a taboo” and is now significantly more “acceptable” (Int. 28) than in previous times:

But everyone knows from their circle of friends and family how quickly something like this can happen. And then even the strongest, most resilient person suddenly feels drained. They have problems, which may not necessarily be depression. They may just be in a depressed mood or something similar. And that’s why I hope that we all realize that it’s not just some weakling who somehow gets sick, but any one of us (Int. 25).

Employers point to social and workplace conditions that they describe as harmful to health, such as increasing workforce demands, faster work pace, and heavier workloads:

There’s just more of, let’s say, burnout. However that manifests itself. We’re seeing more of that too, along with overload. You can just tell, even without having a specific case. But when I think about our workplace or even the last few years, the pressure is definitely increasing. More is expected of everyone. Professionally, privately, and you can really feel it now (Int. 5).

This is also linked, in part, to criticism of inadequate healthcare, particularly in the area of mental health, which leads to long waiting times and a lack of support.

#### Uncertainties in dealing with mental health issues

In addition to the normalisation described above, employers express a variety of uncertainties. These concerns relate both to the extent of mental health challenges within their own organisations and to how to specifically support affected employees. Several respondents assume that there are employees with mental health issues in their companies, even if this is not openly discussed:

I think, as far as I can tell, that it… always depends. I’m sure there are employees in our company who may have a mental illness that we know nothing about. I’m certain of that. With two thousand people, that’s one hundred percent guaranteed. So there are definitely employees who may be undergoing treatment. In whatever form, or for whatever reason (Int. 5).

Uncertainty in dealing with mental illness is also reflected in the fact that such conditions are sometimes described as difficult to grasp. This is attributed, on the one hand, to the “invisibility” of mental illness itself, and on the other hand, to the difficulty of distinguishing illness-related behaviors from individual personality traits. This is further compounded by a lack of knowledge regarding diagnoses and the progression of mental illness:

So these are people who are now mentally ill, even if for me in quotation marks, a mental illness is something I can’t really comprehend from a personal perspective. I know that such illnesses exist, but to be honest, it’s a bit incomprehensible to me (Int. 13).

That we as colleagues didn’t notice anything until day X, when the first sick note came in (Int. 19).

#### Different perceptions of diagnoses

In some cases, employers distinguish between different mental health problems and use this distinction to determine how to handle affected employees in the workplace. From the employers’ perspective, “manageability” in the workplace depends on the type and severity of the illness. Depression and burnout, in particular, are framed as “milder” illnesses and are generally considered more manageable:

Depressive disorders are usually manageable, even for the workplace… Often, the same job can be resumed (Int. 3).

“Severe” mental health problems, such as bipolar disorder, borderline personality disorder, or schizophrenia, are described as more difficult to manage because they tend to create uncertainty within the team and are associated with risky behavior or absences that are hard to predict:

What is extremely difficult are, I think, bipolar disorders… these people can sometimes cause collateral damage. In the team, at their workplace, with customers (Int. 3).

So with [employee 1]… with borderline personality disorder… much more difficult… every now and then a few weeks in the clinic (Int. 12).

There was a person who was surely going through some kind of schizophrenic phase… everyone was a little afraid (Int. 8).

#### Specific challenges in day-to-day work

In many cases, the uncertainties mentioned relate to their impact on employees’ ability to work and their job performance. Employers are particularly concerned that employees may have limited resilience and take more frequent leave, or that their skills and behavior may not be optimally suited to the demands of the position or the team:

So the issue of shifts is certainly an important one, but also, when we talk about piecework, it means that on the one hand I have to perform well, but on the other hand it’s also the case that other employees are always dependent on my personal work performance. So if they don’t make progress at the right pace, then two or three people have to wait until they’re finally done, and that just creates a lot of stress (Int. 9).

Employers also refer to the unpredictability of managing employees with mental health problems as a challenge. The course of mental health problems is described as difficult to predict, both in terms of absenteeism and the impact on employees’ continued ability to work, for example following a prolonged period of absence. Some reintegration processes or clarifications with insurance providers often take months or even years and require a significant investment of time and organisational resources. Employers also note that the individual resilience of those affected often changes on short notice, making reliable staffing and scheduling difficult.

You can’t just say, I’ll hire someone to replace him. You don’t know, maybe he’ll be back tomorrow. But the day after tomorrow, maybe he won’t be there again because he just can’t get out of bed (Int. 20).

In addition, employers express concerns about dealing with employees with mental health problems that go beyond the immediate impact on their work and primarily affect the team. Specific concerns include, for example, behavior that violates boundaries in the workplace (e.g. office rules), uncertainty about how to interact with affected colleagues, and worries and fears:

When someone behaves in such a way that colleagues say, I’m not working with him anymore. I’m afraid of him (Int. 9).

Employers invoke the principle of “fairness” in their explanations by weighing the needs of individuals (those affected) against those of the entire team:

Is the individual relevant or the collective? I think that in some cases this can also lead to a split in a team, because the others simply say: Listen, with all due respect to your illness, I’m important too (Int. 18).

### Between social responsibility and pragmatism: supportive attitudes among employers

Employers demonstrate a variety of supportive and resource-oriented attitudes toward the employment of workers with mental health problems. These relate in particular to 1. assuming social responsibility, 2. viewing experiences of mental health crises as a resource, 3. pragmatic considerations, and the role of 4. positive prior experiences.

#### Social responsibility

Social responsibility appears to be a central motive for employers to hire people with mental health problems and to support them accordingly in their daily work. In some cases, social commitment is justified by personal experience with crisis, e.g., within one’s family or circle of friends. An expressed empathetic attitude is also linked to the desire to give employees with (mental) disabilities a chance:

In principle, everyone deserves equal opportunities. So, whether you have a chronic illness or some other disability. I think everyone should get a chance (Int. 14).

I’ve always seen it as giving people opportunities. So for me, that’s still an inner motivation to this day. You want to give people opportunities (Int. 5).

It is also important to me that these people, with their mental illnesses, are taken seriously in their working lives and can be integrated as much as possible (Int. 19).

The aspect of employers’ social responsibility can, on the one hand, be linked to the aforementioned normalisation or widespread prevalence of mental illness. On the other hand, it also draws connections to the health-promoting aspects of gainful employment, such as providing daily structure, fostering social integration, or boosting self-esteem:

That they belong somewhere. That they have a daily structure. That they don’t just sit at home with all their problems… but have an environment where they feel their own worth (Int. 4).

#### Experiences of mental health crises as a resource

Employers also view experiences of mental health crises as a valuable resource for the workplace. From this perspective, successfully managing or overcoming a mental health crisis is seen as a positive trait that can be leveraged as a skill in the workplace:

I also conclude from this to a certain extent that there is a corresponding level of self-confidence, enabling one to deal reasonably well with other problems and to address them openly (Int. 7).

Because he of course has an employee who has experience with mental illness or impairment, and therefore a different approach to patients with similar problems (Int. 8).

In this regard, some employers point out that experiencing a mental health crisis can have a positive impact on personal development, foster self-confidence, or increase sensitivity, e.g. to the need for setting boundaries:

For me, it’s always a huge achievement when they try to overcome an addiction or mental illness to such an extent that they can then lead a completely normal, self-reliant, and resilient working life (Int. 3).

But that perhaps completely different strengths can also be developed if someone is more sensitive, if someone is perhaps more aware of their limits than others who are not yet aware of them (Int. 6).

If successfully coping with crisis situations is viewed as an additional skill, this can help qualify employees for more advanced roles. For example, one participant reported that positive experiences with a colleague who had previously been affected by a crisis ultimately also contributed to her professional development within the organisation:

It was such a positive experience that we subsequently promoted another employee, who had grown up with the company and had previously undergone therapy there, to facility manager (Int. 2).

#### Labor shortages and economic efficiency

Another reason employers hire people with mental health problems stems from structural factors in the labor market. Against the backdrop of a growing shortage of skilled workers, employees are viewed as valuable, regardless of their mental health:

My motivation isn’t because I’m a do-gooder, but because I can’t find any workers. I simply can’t find any workers (Int. 7).

Promoting workforce participation goes hand in hand with employers’ economic interests:

They have also performed flawlessly and achieved a great deal over a long period of time, and someone who is trained and well qualified is someone you want to continue employing once they have made a full or partial recovery. Of course, it’s also more economical (Int. 27).

I think everyone is important per se. Otherwise, it would be bad if I were a human resources manager. And everyone actually has skills that can be put to use. For the collective. And it’s just a question of to what extent, what skills you have at that moment, and how you can use them. Apart from that, we have enough work to be done on a practical level, and everyone who works with us is welcome and necessary (Int. 18).

#### Previous positive experiences

Previous positive experiences, such as successful or uncomplicated processes, have a positive impact on employers’ actual willingness to hire employees with mental health problems:

From the very beginning, that was where I said: It just went well (Int. 7).

### Between support and personal responsibility: conditions for successful collaboration

Employers outline specific expectations for employees with mental health problems. These expectations focus in particular on 1. transparency and self-assessment, 2. personal responsibility, and 3. disclosure as the foundation for workplace support and mutual trust.

#### Transparency and self-assessment

Employers emphasize that, when managing workplace situations, they focus less on the formal diagnosis and more on the practical implications of the condition for day-to-day work. Employers want a realistic assessment and a transparent approach to addressing individual limitations or support needs:

It would be enough to know what the diagnosis is and what it means for him in everyday life. Where do you struggle? Where do you reach your limits? Where do you have problems in communication or in interacting with others? (Int. 20).

#### Initiative and responsibility

Another expectation employers have of their employees concerns their proactive approach to mental health and their active efforts to seek treatment for the condition:

In my personal opinion, the applicant should have already taken steps to try to stabilize his mental health. So that we have a basis on which we know: ‘Okay, he’s taking care of it personally’ (Int. 22).

The responsibility for maintaining mental health is primarily seen as lying with the individual concerned, while workplace support services are viewed as supplementary. The emphasis on personal initiative as a key prerequisite for successful collaboration is also reflected in a critical distinction from the expectation that employers are primarily responsible for the well-being of their employees:

This attitude of entitlement, like, ‘Hey, boss, you do it. You make sure I’m happy (Int. 9).

### Disclosure

Employers emphasize that workplace support is only possible if there is sufficient information about specific needs in the workplace. In this context, disclosing mental health problems is described as a key prerequisite for employers‘ appropriate support:

As employers, we cannot respond if we don’t know what we need to respond to. So openness is actually the most important thing of all (Int. 18).

Employers are considering the potential negative consequences of (full) disclosure of mental health problems in the workplace, particularly with regard to possible stigmatisation. When weighing the pros and cons, however, the risks of non-disclosure are considered more serious. Particular emphasis is placed on a lack of understanding from employers and co-workers and thus, the difficulty of offering adequate support. From the employers’ perspective, without the necessary information, support services cannot be initiated or workplace accommodations made.

### Trust as a prerequisite and consequence of openness

Disclosure is described as being subject to specific conditions: Employers point out that trust in supervisors or colleagues is a key prerequisite for sharing information about one’s mental health or personal details with others:

So, of course, you have to feel confident in your surroundings before you open up a little and perhaps say something that isn’t something you usually like to talk about or hint at (Int. 12).

At the same time, an open approach to individual limitations and needs (see 3.1) can also help foster a relationship of trust between supervisors and employees, thereby creating the conditions for appropriate support, for example, in the form of workplace accommodations or greater understanding within the team.

## Discussion

This study examined employers’ perspectives on the workplace inclusion of people with mental health problems and highlights the ambivalent ways in which inclusion is understood in organisational contexts. While employers expressed supportive orientations and emphasised the importance of social participation, these perspectives were accompanied by concerns about practical feasibility, team functioning, and the organisation of everyday work processes. While such concerns resonate with established findings on stigma-related barriers in employment contexts [8, 9], the present results suggest that these concerns were also expressed in relation to practical aspects of work organisation, team functioning, and perceived feasibility of support. Employers’ accounts reflected a tension between support for inclusion and uncertainty about its practical implications for everyday work.

They described an increasing normalisation of mental health problems in both societal and workplace contexts, echoing previous research documenting growing awareness and changing public discourses on mental health [14]. However, the findings suggest that growing awareness of mental health issues in society and organisations does not necessarily translate into greater confidence in dealing with employees. Although mental health problems were described as increasingly “normal”, employers continued to refer to unpredictability, fluctuating work capacity, and potential disruptions to established workflows. Similar tensions have been observed in previous qualitative studies, which point to the coexistence of inclusive intentions and perceived organisational constraints [16, 18].

Employers’ differentiation between diagnostic categories further illustrates how inclusion is evaluated in relation to perceived manageability. Depression was often framed as compatible with workplace demands, whereas psychotic or personality-related conditions were more frequently associated with uncertainty or risk. Such distinctions correspond with research on uneven mental health literacy [20] and continuing discrimination towards people with severe mental illnesses [9].

Alongside these concerns, employers expressed a range of facilitating orientations. Social responsibility and the perceived value of employment for recovery were mentioned, echoing evidence on the health-promoting effects of work [2]. Beyond employment itself, recent findings indicate that perceived workplace inclusion is associated with favourable outcomes for health, well-being, and productivity among employees with chronic health impairments [21]. In addition, pragmatic considerations, including labour shortages and the retention of experienced staff were described as relevant factors in decisions to support employees with mental health problems. Similar combinations of social and pragmatic motives have been reported in studies on inclusive employment practices [22]. Positive prior experiences also appeared influential, consistent with research suggesting that contact can influence subsequent attitudes in both inclusive and exclusionary directions [23, 24].

A particularly salient finding concerns the conditional character attributed to workplace participation. Employers frequently linked inclusion to expectations regarding transparency, individual responsibility, and disclosure. While the literature has predominantly addressed disclosure as a dilemma for employees due to the risk of stigma [25, 26], the present findings indicate that employers often framed disclosure as necessary for anticipating support needs and maintaining predictability within organisational processes. This perspective resonates with qualitative work emphasising the relational and context-dependent nature of disclosure decisions [27], while also pointing to the organisational functions disclosure may serve.

The emphasis on trust further underlines that inclusion was described as a relational process. Employers referred to supportive organisational cultures and stable supervisory relationships as key conditions for open communication, reflecting broader findings on the role of workplace climate in shaping inclusion outcomes [14]. This is also consistent with recent findings showing that organisational conditions, particularly an appreciative and safe team climate, play an important role in employees’ experiences of workplace inclusion [28]. At the same time, expectations of openness may place additional demands on employees, particularly in contexts where power asymmetries and stigma-related risks remain salient.

Overall, the findings suggest that employers’ perspectives on workplace inclusion reflect both inclusion-related values and practical organisational considerations. These perspectives appeared to be grounded in prior experiences, situational demands and broader societal discourses on mental health.

### Strengths and limitations

A strength of the study lies in its qualitative and participatory design, which enabled an in-depth exploration of employers’ perspectives and the organisational contexts shaping workplace inclusion. The inclusion of decision-makers from different sectors provided insights into diverse organisational experiences and considerations. However, the findings should be interpreted in light of certain limitations. The sample was regionally confined and may not capture variations across broader labour market or institutional contexts. Participation may also have been influenced by self-selection, potentially favouring employers with greater interest in mental health topics. Moreover, the study is based on employers’ accounts and does not provide direct insight into everyday workplace practices or how these perspectives are enacted in concrete organisational settings. As the focus was on employers, the findings do not reflect how these expectations and interpretations are experienced or negotiated by employees themselves. Finally, given the exploratory qualitative design, the study was not intended to quantify the prevalence of specific perspectives or systematically compare subgroups of employers. Potential distribution patterns and group differences are addressed in the subsequent quantitative phase of the mixed-methods study.

## Conclusions

This study provides insights into how employers make sense of the workplace inclusion of people with mental health problems within the constraints of everyday organisational practice. Employers’ perspectives were characterised by ambivalence, reflecting efforts to balance normative commitments to participation with concerns related to predictability, fairness, and collective functioning. While supportive orientations were evident, inclusion was often described as contingent upon organisational feasibility and shaped by expectations regarding transparency, responsibility, and trust.

The findings suggest that employers’ perspectives on workplace inclusion were related not only to individual attitudes, but also to organisational considerations, prior experiences, and broader understandings of mental health. In this context, expectations surrounding disclosure and self-management appeared closely linked to attempts to manage uncertainty and coordinate work processes. Recognising these dynamics may help to move beyond approaches that focus exclusively on stigma reduction and towards strategies that also address organisational conditions shaping participation. Future research could further explore how these organisational processes unfold across different sectors and institutional contexts, as well as how employees themselves experience and negotiate the expectations described by employers. 

## Supplementary Information

Below is the link to the electronic supplementary material.

Supplementary Material 1

## Data Availability

The datasets generated and analysed during the current study are not publicly available due to the sensitive nature of qualitative data and the potential risk of participant identification but are available from the corresponding author on reasonable request.

## Abbreviations

BMAS: German Federal Ministry of Labour and Social Affairs (Bundesministerium für Arbeit und Soziales)
COREQ: Consolidated Criteria for Reporting Qualitative Research
FIS: Interdisciplinary Social Policy Research Funding Network (Fördernetzwerk Interdisziplinäre Sozialpolitikforschung)
SMI: Severe Mental Illness
TAPE: Promoting the participation of people with severe mental illness in the workplace. Attitudes, barriers, and facilitating factors (Teilhabe auf dem Allgemeinen Arbeitsmarkt bei Psychischen Erkrankungen)
