# Supplementary material for: Between support and uncertainty: employers’ perspectives on workplace inclusion of people with mental health problems
Source: BMC Health Serv Res. 2026 Sep 28;26:1321. doi: 10.1186/s12913-026-15665-x (PMC13621519; doi:10.1186/s12913-026-15665-x)
Supplement: Supplementary file 1 [file 12913_2026_15665_MOESM1_ESM.docx]

***TAPE study***

Thank you very much for your interest in our study. First, I would like to provide you with some information about the procedure of today’s interview.

I would like to ask some questions about your experiences with employing people with mental illnesses, as well as barriers and facilitating factors regarding the employment of people with mental illnesses in the primary labor market. We are particularly interested in your experiences with successful workplace design for participation and effective forms of support.

Presumably, the interview will take approximately 45–60 minutes. However, we will adapt this entirely to your pace. You may answer each question in as much detail as you like. If anything is unclear, please feel free to ask at any time.

**Interview Guide for Interviews with Employers with experiences in employing people with mental health problems**

| Topic | **Guiding Question** | **Follow-up questions (optional)** | **Optional follow-up questions** |
| --- | --- | --- | --- |
| Introduction | **To begin with, I would be interested in why you agreed to participate in an interview on this topic.** |  |  |
| Previous Experiences | **What are your experiences with employing people with mental illnesses?**  **What role does the mental illness specifically play in everyday working life?**  **Was there anything that surprised you, compared to your previous expectations?** | How did it come about that you hired this person?  What would you say: overall, how did it go? | Can you tell me a bit more about that? |
| Motivation | **What is your motivation for employing people with mental illnesses?** | What role do…  …… operational conditions/reasons play? (structure, tasks, employees)  … the person’s own characteristics or abilities play? (competencies, qualifications, experiences)  … conditions outside the company play?  … societal reasons play? | Do you have a specific example of this?  Could you explain that in a bit more detail? |
| Disclosure in the workplace context | **What role does the disclosure of the mental illness at the workplace play?** | How openly should mental illness be addressed in the workplace? What should be disclosed?  How was the disclosure handled? What did the disclosure mean to you? **How did it affect your view of the person?**  How did the environment react to the disclosure? What changed in the environment? |  |
| Hindering Factors  *Important: From the perspective of companies* | In practice, it becomes apparent that the employment of people with mental illnesses also depends on conditions within the company.  To what extent is the company capable of responding adequately to the needs of the employees? | What possibilities do you see? Are there also limitations?  In cases of negative experiences:  Were there any further difficulties? What were they specifically?  What does this mean for everyday working life?  …for the person themselves? (characteristics and abilities)  …for the company?  …for the team?  How did you deal with the difficulties?  …at management level?  …in communication with the person?  …within the team?  …any other persons involved, e.g. external ones?  What would you have wished for, or what would have been needed to improve the situation? | Do you have a specific example of this?  Could you explain that in a bit more detail? |
| Facilitating Factors  *Important: From the perspective of companies* | **What is helpful for you as a company when employing people with mental illnesses?** | In cases of positive experiences:  What contributed to things going so well?  What works particularly well in everyday working life?  How was this handled within the team of colleagues?  Support from the organisational environment  What kind of support do supervisors and colleagues in the company need?  Collaboration  How can communication between all parties involved (employers, colleagues, individuals concerned, job coaches) be best organised? What is helpful in this regard?  Forms of support  Support can be helpful for companies in the specific phases of employment, i.e. during entry, induction, and maintenance of employment. This support can be provided both within and outside the company.  Which forms of support do you consider useful?  How can they be provided sustainably? And who could be responsible for this?  Making good practice examples visible  You have already reported positive experiences regarding workplace design for people with mental illnesses.  OR: Other companies have reported positive experiences regarding workplace design for people with mental illnesses. How can these experiences be made transparent and usable for other companies? | What helped you in this situation? What was helpful?  How did you resolve the situation? (active role of the interviewee)  Can you give an example?  How did you determine that?  Follow-up questions on forms of support  Are there any additional in-company forms of support that you consider useful?  Are there any additional external forms of support that you consider useful?  Do you have any further ideas regarding entry/induction/maintenance of employment? |
| Conclusion and Thank You |  | We have now reached the end of the interview: Is there anything that we have not discussed yet, but that you would like to mention?  Thank you very much for your participation! |  |

**Interview Guide for Interviews with Employers without experiences in employing people with mental health problems**

| Topic | **Guiding Question** | **Follow-up questions** | **Optional follow-up questions** |
| --- | --- | --- | --- |
| Introduction | **To begin with, I would be interested in hearing why you agreed to participate in an interview on this topic.** |  |  |
|  | **What role does mental health play in your company?**  **To what extent are mental illnesses an issue in your company?** | How is long-term absence handled? Is there an occupational reintegration management system in place? How does it work?  What expectations or assumptions do you have regarding employees with mental illnesses?  Have you had any experience with potential employees with mental illnesses? Would you be willing to employ people with mental illnesses?  What do you think: If a person in your company were mentally ill, would you be aware of it?  Where would a person with a mental illness turn to for support?  How would the team deal with it if a colleague became mentally ill?  What impact would a mental illness have on the company? | Could you please explain that in a bit more detail?  How exactly did that happen?  And what happened next?  How was it handled? |
| Motivation / Hindering Factors | **What currently prevents you from employing people with mental illnesses in your company?** | What role do …  … organizational conditions play? (structure, tasks, employees)  … the person’s own characteristics or abilities play? (competencies, qualifications, experience)  … external conditions play? | Do you have a specific example of this?  Could you explain that in a bit more detail? |
| Disclosure in the workplace context | **What role does the disclosure of a mental illness play?** | How openly should mental illness be addressed at work? What should be disclosed?  How should disclosure be handled? What would disclosure mean for you? What would it mean for your perception of the person?  How would the work environment react to the disclosure? |  |
| Facilitating Factors  *Important: From the perspective of companies* | **From your perspective, what would specifically be needed to employ people with mental illnesses?** | … regarding the person?  … regarding your company?  … regarding conditions outside your company?  What would specifically support you in this context?  e.g. internal: which people, institutions  e.g. external: which people, institutions  What else would be helpful for you? |  |
| Making Good Examples Visible | **Companies have reported positive experiences with workplace design for people with mental illnesses.**  **What do you think might be special or different in these companies?** | How can these experiences be made transparent and usable for your company or for other companies? |  |
| Conclusion and Thank You |  | We have now reached the end of the interview: Is there anything that we have not discussed yet, but that you would like to mention?  Thank you very much for your participation! |  |
